# Recovery of Recurrent Parent Genome in a Marker-Assisted Backcrossing Against Rice Blast and Blight Infections Using Functional Markers and SSRs

**DOI:** 10.3390/plants9111411

**Published:** 2020-10-22

**Authors:** Samuel Chibuike Chukwu, Mohd Y. Rafii, Shairul Izan Ramlee, Siti Izera Ismail, Yusuff Oladosu, Isma’ila Muhammad, Ibrahim Musa, Muideen Ahmed, Muhammed Itopa Jatto, Bashir Rini Yusuf

**Affiliations:** 1 Laboratory of Climate Smart Food Crop Production, Institute of Tropical Agriculture and Food Security, Universiti Putra Malaysia, UPM Serdang, Selangor 43400, Malaysia; chukwu.samuel@ebsu.edu.ng (S.C.C.); oladosuy@upm.edu.my (Y.O.); ismuha@gsu.edu.ng (I.M.); ibrahimmusa@fukashere.edu.ng (I.M.); gs47922@student.upm.edu.my (M.A.); gs51856@student.upm.edu.my (M.I.J.); bashir.yrini@fugusau.edu.ng (B.R.Y.); 2 Department of Crop Production and Landscape Management, Faculty of Agriculture and Natural Resources Management, Ebonyi State University, Abakaliki PMB 053, Nigeria; 3 Department of Crop Science, Faculty of Agriculture, Universiti Putra Malaysia, UPM Serdang, Selangor 43400, Malaysia; shairul@upm.edu.my; 4 Department of Plant Protection, Faculty of Agriculture, Universiti Putra Malaysia, UPM Serdang, Selangor 43400, Malaysia; izera@upm.edu.my

**Keywords:** *Oryza sativa* L., chromosome, introgression, microsatellites, foreground selection, background selection, RPGR

## Abstract

The most vital aspect of marker-assisted backcross breeding is the recurrent parent genome recovery. This enables the selection of only parents with recovered recipient/recurrent parent genome in addition to the targeted genes. The recurrent parent genome recovery (RPGR) ensures that non-desirable genomic segments are removed while the gene of interest is sustained in the recombined progenies without further segregations. This work was aimed at quantifying the RPGR of backcross populations with introgression of bacterial leaf blight resistance genes. Putra-1, a Malaysian elite variety, high yielding with inherent resistance to blast but susceptible to bacterial leaf blight (BLB), was crossed with IRBB60 which is resistant to BLB disease. The IRBB60 has four *Xoo* resistance genes—*Xa4*, *xa5*, *xa13* and *Xa21*. Tightly linked polymorphic functional and SSR markers were used for foreground selection at every stage of backcrossing to select progenies with introgressed target genes. Background selection was done to quantify the percentage of RPGR in the selected lines using 79 confirmed polymorphic microsatellites. Result obtained showed that the percentage of RPGR was 80.11% at BC_1_F_1_, 95.30% at BC_2_F_1_ and 95.9% at BC_2_F_2_. The introgression of *Xa4*, *xa5*, *xa13* and *Xa21 Xoo* resistance genes were faster through the adopted marker-assisted backcross breeding compared to what could be obtained through conventional breeding. All the 16 selected lines displayed resistance to BLB with three lines showing high resistance to the disease. The blast resistance contained in the genetic background of Putra-1 was also sustained in all the selected lines. The newly developed lines were recommended as new rice varieties for commercial cultivation.

## 1. Introduction

Rice is an important cereal crop that plays a critical role in human diet [1,2]. Most rice production takes place in the Asian continent with over 150 million ha cultivated all over the world [1,3]. Given a favorable environmental condition, the rice crops would grow fast and produce high yields [4]. The crop requires minimal fertilizer application. It does well in saline water with a high enough quantity of micronutrients. The availability of irrigation facilities and its further expansion, provision of subsidy for machineries, fertilizer, seeds, irrigation, as well as new technologies would lead to increased productivity of rice in various agricultural areas [5]. Singh et al. [6] reported that there will be over eight billion people in the world by 2030 and the population is further predicted to reach nine billion by 2050. The growth in population would require increase of 40% in rice production in order to avert hunger. Blast and blight are fungal and bacterial infections, respectively. Both diseases are very destructive to rice production in several rice agro-ecologies round the world. The diseases are responsible for significant yield reduction in rice production. Previous research has focused on breeding new varieties that are resistant to blast disease and high yielding, e.g., Putra-1 rice variety. The need for sustainable crop development and resistance to biotic stress caused by blast and blight are essential due to the emergence of new races of the pathogens such as *Xanthomonas oryzae* pv *oryzae* (*Xoo*) responsible for bacterial leaf blight (BLB) and *Magnaporthe oryzae* responsible for rice blast [7]. Following the success recorded from conventional breeding over the years, significant progress has been made to develop suitable cultivars that can resist various types of biotic and abiotic stress that affect rice productivity. The emergence of new biotypes necessitated the pyramiding of various resistance genes into cultivars with good agronomic value to ensure durable resistance. This enables the cultivars to resist attacks from different pathogens as well as survival in unfavorable environmental conditions.

Backcrossing is a conventional means of inserting a specific gene controlling a particular trait into an elite variety. It involves the use of two parents known as donor and recipient. The recipient parent is referred to as recurrent parent when it is used repeatedly in the crossing scheme [7]. A disease resistance gene could be transferred from one cultivar (usually not improved) to another cultivar being an elite variety [8]. Marker-assisted backcross breeding offers an efficient and precise method for breeding that preserves the vital characteristics of the recurrent parent, such as a high yielding trait. The underlying principle of marker-assisted backcrossing is to incorporate the specific gene of interest obtained from the donor into the defined locus of the recurrent parent. Marker-assisted backcrossing reduces linkage drag and helps in recovering the recurrent parent genome while simultaneously reducing the donor parent genome. Useful molecular markers could be functional markers and/or simple sequence repeats (SSR/microsatellite) markers, etc. [9]. Backcross breeding aided by marker technique is useful in incorporating disease resistance in rice without sacrificing its genetic background and this is done by several backcrossing to the recurrent parent. Recovery of the recurrent parent genome could be achieved using marker-assisted background selection. However, the high cost of molecular markers and the limitations of microsatellites in detecting polymorphisms, as well as the need for prompt execution of the whole process, are some of the constraints of marker-assisted background selection [10]. Laha et al. [11] also noted that limitations for natural screening of BLB are labor-intensive and time consuming and due to variation in the degree of natural infection. Artificial inoculation of BLB could be the most effective method of screening and it could be performed by a number of strategies such as prick inoculation of the leaves, spraying the plants with bacterial suspension, dipping the seedlings in bacterial suspension before transplanting and cutting the leaves and then spraying with bacterial suspension.

Marker-assisted backcrossing involves the use of marker to select for the target locus and subsequently enhance the recovery of recurrent parent genome [12]. On this basis, marker-assisted backcross breeding basically consists of three levels known as the foreground, background and recombinant selections [13]. The background selection accelerates the recurrent parent genome recovery (RPGR) ratio, thereby saving the breeder some cycles of selection [7]. At every stage of backcrossing, the proportion of donor parent genome is brought down by half. Hence, the RPGR percentage is expressed as a ratio to the donor parent genome recovery percentage [5,8]. Recurrent parent marker alleles could be used to select all genomic regions during background selection and target locus could also be selected using phenotypic screening. Background selection facilitates the recovery of recurrent parent genome during marker-assisted backcross breeding while further backcrossing leads to varietal development and conversion of complete line [14,15]. Therefore, the objective of this study was to quantify the recurrent parent genome recovery of the new introgression lines from the cross Putra-1 × IRBB60 using functional and SSR markers.

## 2. Results

### 2.1. Foreground Selection of F_1_ Hybrids and Backcross Populations

**Figure 1:**
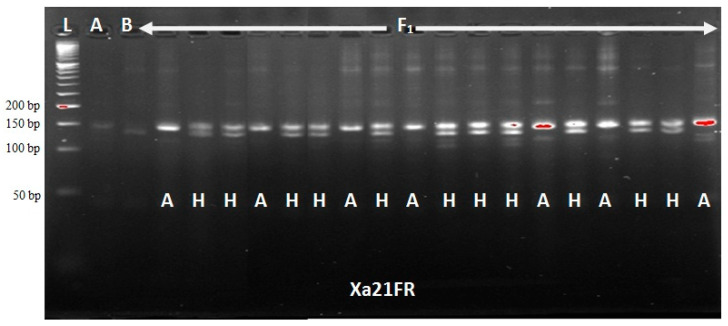
Foreground selection of F_1_ hybrids using Xa21FR functional marker.

The functional marker Xa21FR revealed a total of 46 true F_1_ plants with heterozygous alleles from a list of 72 F_1_ plants produced (Figure 1). However, all the blast resistance genes, *Piz*, *Pi2* and *Pi9*, and bacterial leaf blight R-genes, *Xa21*, *xa13*, *xa5* and *Xa4,* were confirmed in only five F_1_ plants and were selected as hybrids for use in backcrossing. At BC_1_F_1_, a total of 108 plants out of 288 produced were confirmed to carry *Xa21* BLB R-gene. Additionally, *xa13*, *xa5* and *Xa4* BLB R-genes were incorporated into 125, 118 and 122 plants, respectively. On the other hand, the blast resistance genes were confirmed in 112 plants only. The result obtained on *Chi*-square (χ^2^) analysis indicated a goodness of fit (no significant difference) to 1:1 Mendel’s segregation ratio (single gene model) for foreground markers at BC_1_F_1_ (Table 1). Selection at BC_1_F_1_ was made on nine plants only that were confirmed to carry all BLB and blast resistance genes studied, and were used for subsequent backcrossing. At BC_2_F_1_, 106 heterozygous plants out of a total of 268 produced were confirmed to carry dominant *Xa21* and recessive *xa13* genes each, using the functional markers Xa21FR and Xa13prom, respectively. In the same way, *xa5* and *Xa4* were confirmed present in 106 and 96 plants, respectively. The blast resistance genes were confirmed in 108 plants only, using the SSR markers RM6836 and RM8225. Both blast and BLB R-genes were found to be incorporated in 14 progenies only at BC_2_F_1_. Background selection revealed only nine out of the 14 plants to have sufficiently recovered their recurrent parent genome and these were selected for the next stage of crossing. A goodness of fit to a 1:1 Mendel’s ratio for a single gene model was obtained using chi-square test (Table 2).

**Figure 2:**
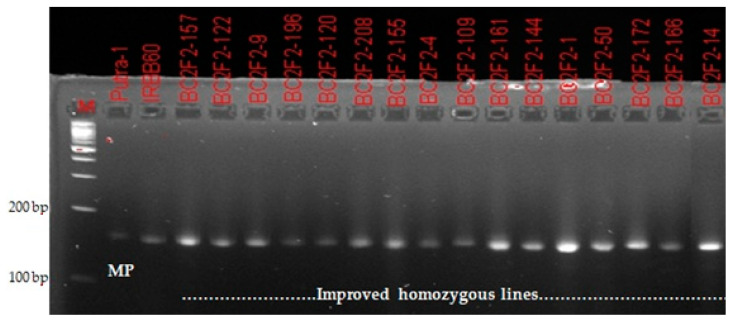
Confirmation of bacterial leaf blight resistance in homozygous improved lines using the functional marker MP.

**Figure 3:**
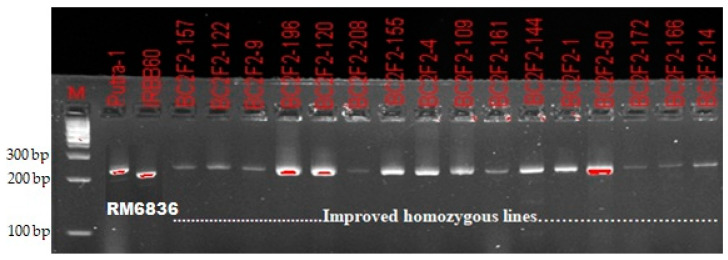
Confirmation of blast resistance in homozygous improved lines using the SSR marker RM6836.

A total of 220 BC_2_F_2_ plants were grown from nine selected recurrent parent genome recovered BC_2_F_1_ lines. The result obtained from molecular genotyping showed that *Xa21* gene was fixed in 17 plants. Additionally, homozygous alleles for *xa13, xa5* and *Xa4* BLB resistance genes were confirmed in 32, 17 and 115 plants, respectively. For blast resistance genes, homozygous alleles similar to that of recurrent parent (Putra-1) were confirmed in 69 plants only. Final selection was made from homozygous individuals carrying the donor (IRBB60) parent allele with high RPGR percentage (Figure 2 and Figure 3). Contrary to the BC_2_F_2_result for *Xoo* resistance, the ratio of 69:100:49 obtained for blast resistance marker segregation using chi-square analysis indicated a goodness of fit to the Mendelian expected 1:2:1 segregation ratio at BC_2_F_2_ (Table 3).

### 2.2. Marker-Assisted Background Selection of Backcross Populations

Out of the nine progenies assessed for RPGR at BC_1_F_1_, six individuals (BC_1_F_1_-38, BC_1_F_1_-328, BC_1_F_1_-15, BC_1_F_1_-36, BC_1_F_1_-97 and BC_1_F_1_-105) with a minimum of 80% (mean recorded) RPGR and above were selected and these individuals were preferred for BC_2_F_1_ crossing [16,17]. The best progeny in BC_1_F_1_ population was BC_1_F_1_-38, with an RPGR of 86.40%, a low heterozygous component of 8.70% and a reduced donor genome of 4.90%, in addition to very negligible linkage drag. With the result obtained on RPGR from marker-assisted background selection of BC_2_F_1_ after genotyping, coupled with further confirmation through phenotyping, the nine best BC_2_F_1_ progenies with minimum RPGR of 95.31% (mean recorded) were selected. These nine best BC_2_F_1_ were chosen as recombinant parental seeds and selfed to produce BC_2_F_2_ generation.

### 2.3. Recurrent Parent Genome Recovery of the Selected Improved BC_2_F_2_ Lines

**Figure 4:**
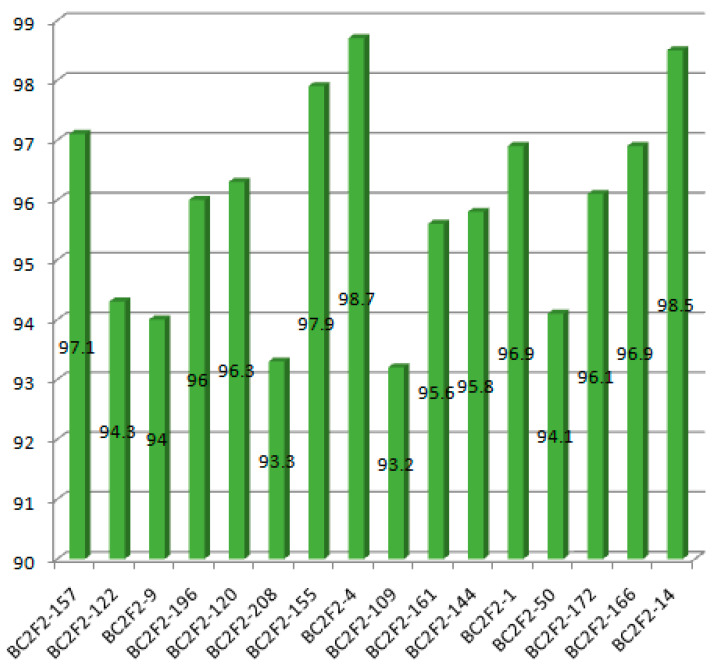
Recurrent parent genome recovery percentage of the improved selected lines.

**Figure 5:**
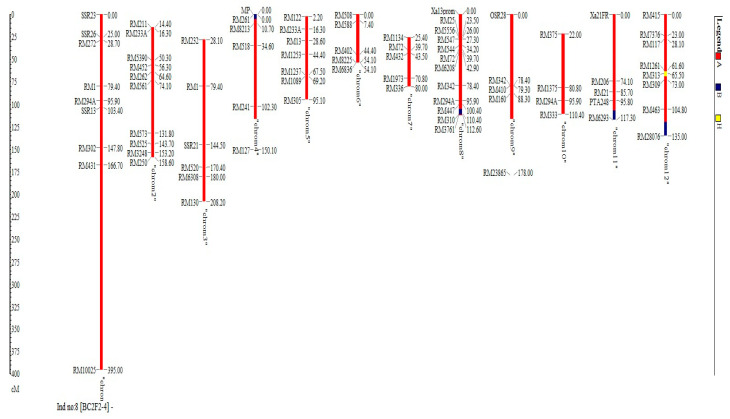
Highest chromosome-wise recurrent parent genome recovery of the improved selected lines observed in BC_2_F_2_-4.

The result shows that the RPGR obtained at BC_2_F_2_ranged from 93.2% to 98.7% (Figure 4). The line BC_2_F_2_–4 recorded the highest RPGR. The 16 selected lines had average RPGR of 95.9% spread across the 12 chromosomes. The percentage of the donor parent genome ranged from 0.2% in BC_2_F_2_–50 to 4.3% in BC_2_F_2_–122. The mean proportion of donor parent genome recorded was 1.7%. Additionally, the proportion of heterozygous genome ranged from 0.7% in BC_2_F_2_–161 to 5.6% in BC_2_F_2_–50. This result indicated that recombination after one generation of self-fertilization from BC_2_F_1_ to BC_2_F_2_ resulted to 0.75% increase in recurrent parent genome recovery, 0.26% reduction of the donor parent genome and 0.50% reduction in the heterozygous genome proportion. The highest chromosome-wise RPGR of the improved selected lines observed in the lines BC_2_F_2_–4 is as shown in Figure 5.

### 2.4. Genetic Increase of the Recurrent Parent Genome Size in Backcross Generations

The size of recurrent parent genome recorded in BC_1_F_1_ ranged from 1198.2 cM to 1566.2 cM while it ranged from 1441.5 cM to 1566.2 cM at BC_2_F_1_ generation. However, the mean recurrent parent genome size ranged from 1307.1 cM to 1510.7 cM in BC_1_F_1_ and BC_2_F_1_, respectively. This is an increase of 203.6 cM after the two successive backcross generations. Additionally, the individual with the highest RPGR at BC_1_F_1_ generation (BC_1_F_1_-38) recorded a recurrent parent genome size of 1373.9 cM, while the individual with the highest RPGR at BC_2_F_1_ (BC_2_F_1_-81) had a recurrent parent genome size of 1566.2 cM. This indicated a genetic increase in the recurrent parent genome size of 192.3 cM. These results unveil the potentials of molecular marker-assisted backcross breeding in the progressive restoration of recurrent parent genome size in backcross populations [18].

### 2.5. Genetic Decrease of the Donor Parent Genome Size in Backcross Generations

The genome size of the heterozygous segment ranged from 7.5 cM (BC_1_F_1_-105) to 295.7 cM (BC_1_F_1_-521) at BC_1_F_1_ generation, while it ranged from 7.5 cM (BC_2_F_1_-81) to 116.8 cM (BC_2_F_1_-198) at BC_2_F_1_ generation. The average heterozygous genome size was 150.9 cM and 42.1 cM at BC_1_F_1_ and BC_2_F_1_ generations, respectively. Additionally, the individual with the highest RPGR at BC_1_F_1_ generation recorded heterozygous genome size of 138.8 cM, while the individual with highest RPGR at BC_2_F_1_had a heterozygous genome size 7.5 cM. These results showed reduction in heterozygous genome size from BC_1_F_1_ to BC_2_F_1_. When the result obtained from the donor genome size is critically looked at, a similar trend of decrease was observed. The donor parent genome size ranged from 17.3 cM to 294.5 cM and 3.8 cM to 118.7 cM at BC_1_F_1_ and BC_2_F_1_, respectively. Mean donor parent genome sizes were 132.98 cM and 38.22 cM at BC_1_F_1_ and BC_2_F_1_ generations, respectively. The best progeny at BC_1_F_1_ recorded 78.3 cM, while the best individual at BC_2_F_1_ recorded 17.3 cM. The decrease or reduction in donor parent genome size as well as the heterozygous segment as the research progressed from BC_1_F_1_ to BC_2_F_1_ was proof that the essence of backcrossing is to reduce the donor parent genome while the recurrent parent genome is increased or recovered [7,12].

### 2.6. Agronomic Performance of Selected Backcross Lines

The results obtained on the agro-morphological characteristics of the improved selected lines are as presented in Table 4. The mean agro-morphological characteristics of the selected lines recorded were as follows: plant height (110.65cm), flag leaf length to width ratio (13.14), number of panicles per hill (15.94), number of days to flowering (75.75), number of days to maturity (105.8), number of effective tillers (16.06), panicle length (32.64), total number of grains per panicle (177.6), 1000 grain weight (79.45g), total grain weight per hill (52.74g), seed length to width ratio (3.86) and yield per hectare (8.44 t/ha). The result showed that the selected backcross rice lines performed better than their recurrent parents in most traits of agronomic importance. The improved lines differed significantly with their recurrent parent in all the agro-morphological traits studied.

## 3. Discussions

Marker-assisted background selection is useful for obtaining information on the recurrent parent genome recovery. In addition, information pertaining to the donor and heterozygous genome segments are also obtained from background screening. The aim of the plant breeder is to select individuals with the highest recurrent parent genome recovery, as such individuals received the target genes and not sacrificing their recurrent parent genes [19]. Some BC_1_F_1_ and BC_2_F_2_ progenies recorded a lower percentage of recurrent parent genome recovery compared to the theoretical mean obtained at both stages of backcrossing. Similar results have been reported by Neeraja et al. [20] and Yi et al. [21]. Additionally, Sundaram et al. [22] described a “pull” through an unknown mechanism, which could be exercised by the gene of interest in a research project that favored the transmission of additional loci from the donor gene, which would result in a percentage recurrent parent genome recovery that is less than the theoretical mean. However, most of the progenies at both BC_1_F_1_ and BC_2_F_2_met the expected MABB theoretical recurrent parent genome recovery of 79% and 92% at the two backcross generations, respectively [7,9]. The result obtained in this present study corresponds with Sabu et al. [23] and Martinez et al. [24], who reported non-significant difference between the progenies and their parents in grain yield. The number of productive tillers is responsible for the number of panicles obtained in rice [25]. There was a significant increase in panicle length and total number of grains per panicle obtained in this study. These could have contributed to the recovery of grain yield in the improved lines. The number of productive tillers and grain number per panicle have been reported to be associated with high grain yield in rice [26,27]. In the same vein, grain length and width are important quantitative traits that have close relationship with outer physical quality [28,29]. Grain length and width have also been reported to determine the shape of grain/seed [30]. On the other hand, grain shape has been reported by Shi and Zhu [31] to be concurrently influenced by triploid endosperm, maternal and cytoplasmic genes.

The highest proportion of heterozygous genome segment obtained in BC_1_F_1_ was 18.6% (BC_1_F_1_-521) with a recurrent parent genome recovery of 77.9%. The result showed a little background marker deviation of 1.1% from the theoretical 79% expected from MABB. This showed that some of the markers deviated towards the heterozygous genome segment. Lau [17] reported that there could be a preferential inheritance of IRBB60 alleles at some loci that caused the increased heterozygous segment in some progenies. This situation was more prevalent as there were about four plants out of the nine BC_1_F_1_ screened progenies that had more than the mean (10.84%) percentage in the heterozygous genome segment. However, the condition was highly reduced in BC_2_F_1_ generation, as the highest proportion of heterozygous genome segment recorded was 7.3%, while all BC_2_F_1_ progenies met the expected MABB recurrent parent genome recovery of 92.2%. The result of this study was in line with Miah et al. [19], who reported the extent of recurrent parent genome recovery of 75.40–91.3% in BC_1_F_1_ and 80.40–96.7% in BC_2_F_2_ generations. Reflinur et al. [32] observed that the role played by F_1_ either as male or female parent could affect the transmission pattern of alleles. They observed transmission ratio distortion at several loci at BC_1_F_1_ progenies obtained from F_1_ cross that involved *indica* x *japonica* with reciprocal crosses. Where F_1_ played the role of female parent, *japonica* alleles were preferably segregated during F_1_ meiosis at some loci while when backcrossed to *indica*, fertilization between the *japonica* embryo sac and *indica* pollen was highly probable. This resulted in marker segregation that skewed towards the heterozygous genome segment. In the current study, F_1_ and BC_1_F_1_ selected progenies were used as female plants, while Putra-1 served as the males that donated pollens. The high heterozygous genome segment observed in some BC_1_F_1_ progenies was an indication that transmission ratio distortion could have occurred during meiosis. The IRBB60 allele was preferred during meiosis at F_1_ and BC_1_F_1_ which caused more chances of fertilization between the embryo sac that carried the IRBB60 allele and Putra-1 pollen.

Koide et al. [33] described transmission ratio distortion as the preferential transmission of alleles where a pair of alleles is recovered preferentially in the progeny of a heterozygote and such phenomenon causes a deviation in frequency of genotypes expected from Mendelian ratio. Usually, the observed segregation distortion takes place in wide crosses such as the indica x japonica inter-subspecific cross and Basmati x indica inter-group cross [31,34]. Currently, breeders have found the mechanism involved in the transmission ratio distortion of many loci or genes in rice wide crosses. Some examples include sterility gene (*S*) [33,35], gametophytic gene (*ga*) [36] and hybrid breakdown genes (*hbd*) [37].Although some backcross progenies in this breeding program could have suffered from the effects of genes involved in transmission ratio distortion which favored IRBB60 alleles in a backcrossing with Putra-1, not many backcross progenies had high proportion of heterozygous genome segment, especially in BC_2_F_1_. The essence of background selection was to increase the recurrent parent genome recovery and reduce the heterozygous and donor genome segment to a substantial level. The BC_1_F_1_ and BC_2_F_1_ backcross generations could be considered as largely successful, having accelerated the mean recurrent parent genome recovery from a mean of 80.11% at BC_1_F_1_ to 95.3% at BC_2_F_1_. The selected progenies from BC_2_F_1_ population were selfed to produce BC_2_F_2_ seeds, which were planted in the next season to produce BC_2_F_2_ plants. This was essential, because self-fertilization is capable of increasing the homozygosity of non-carrier chromosomes, reduce the heterozygosity and avoid further segregation in subsequent trials [7,38,39].

## 4. Materials and Methods

### 4.1. Source of Germplasm and Breeding Procedure

**Figure 6:**
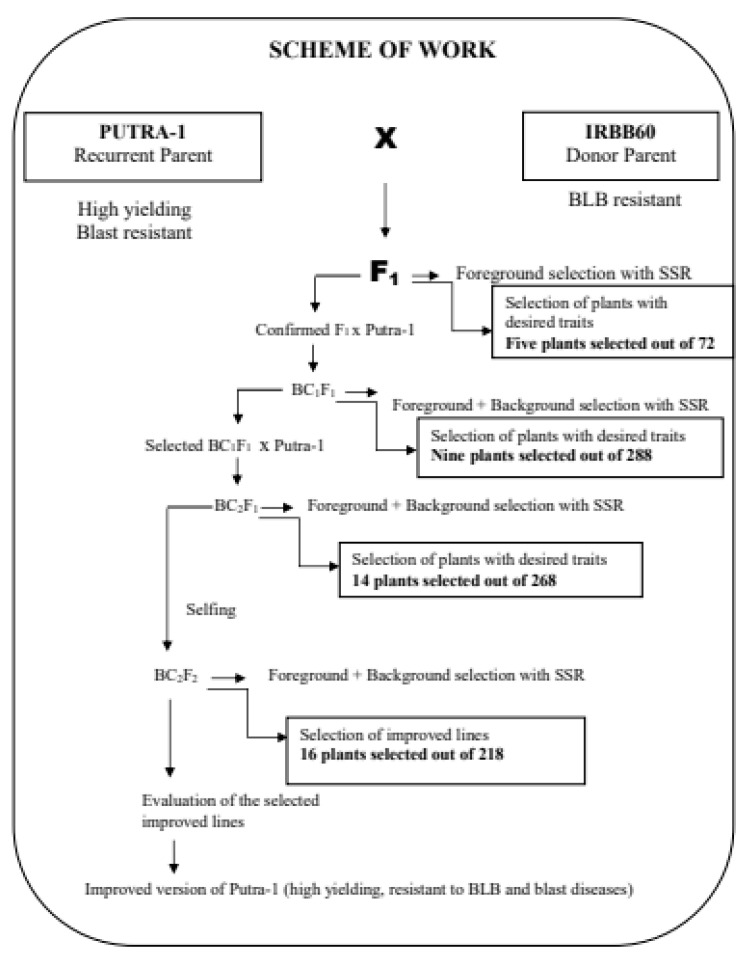
Marker assisted backcross breeding program for blast and blight resistance.

The F_1_ hybrid was developed from a cross between Putra-1 and IRBB60 (Figure 6). Putra-1 had inherent *Pi9, Pi2* and *Piz* blast R-genes, while IRBB60 is an IRRI variety with four *Xoo* R-genes, namely, *Xa21*, *Xa4*, *xa13* and *xa5* [40]. Putra-1 served as female/recipient, and subsequently, the recurrent parent during hybridization and backcrossing, while IRBB60 served as the male parent (donor) during hybridization only, which led to the development of F_1_ plants. True heterozygous F_1_ plants were confirmed using foreground tightly linked functional and SSR markers [40]. Putra-1 was used as recurrent parent in all the backcross generations, with the aim of recovering its high yielding trait. At every stage of backcrossing, the foreground markers which included both functional and SSR markers were used for foreground selection of specific genes of interest. Background selection was carried out with a total of 79 polymorphic SSR markers. Progenies with high recurrent parent genome recovery and reduced donor parent genome segment were selected throughout the breeding program. BC_2_F_2_ plants were selfed to recombine the genes. Figure 6 shows the MABB breeding scheme adopted in the study.

### 4.2. Extraction of DNA and Molecular Marker Screening

After two weeks of transplanting in the glass house, samples of fresh young leaves (0.5 g) were excised from growing plants and subsequently used for genomic DNA extraction. The method of CTAB DNA extraction as proposed by Doyle and Doyle [41] and modified by Ashkani et al. [42] was adopted in this experiment. The DNA concentration, quality and purity were checked with the aid of Nanodrop spectrophotometry machine (Product specification: ND1000 Spectrophotometer USA). The protein contamination level of DNA is represented by the A260/280 ratio, while the A260/230 ratio represents the level of organic contaminants present in the nucleic acid. A 260/280 ratio of ~1.8 is generally accepted as ‘pure’ for DNA, while A260/230 from 2.0 to 2.2 is generally accepted as also ‘pure’ DNA at 230 nm absorbence. However, the most suitable samples of DNA selected for polymerase chain reaction (PCR) were those that had A260/280 purity range from 1.8 to 2.0. The presence of DNA in the extracted sample was also confirmed using gel electrophoresis. From the Image doc result displayed on computer screen, a singular high-molecular-weight DNA band was taken to be good DNA, while smeared and/or multiple allelic DNA bands were considered poor-quality DNA, and hence, not suitable for PCR. The foreground and background markers reported to be associated with resistance against blast and bacterial leaf blight were first screened for polymorphism and suitable ones were selected (Table 5). A mixture of 7.5 uL DNA master mix + 4.5 uL of nuclease free water + 1 uL of forward primer + 1 uL of reverse primer + 1 uL of DNA sample was prepared and spun for thorough mixing using the short spinning machine for 12 s. The PCR mixtures were run for 2.5 hours [10].

### 4.3. DNA Scoring

Based on the banding pattern obtained from the Gel Imager^®^ (GelDocTM XR, Bio-Rad Lab. Inc., Hercules, CA, USA), the progenies were scored with specific reference to their parents. In the banding pattern obtained for the progenies, the progenies that followed the banding pattern of the homozygous recurrent parent was scored as ‘A,’ indicating genotypic resemblance of Putra-1 variety, while progenies that followed the banding pattern of the homozygous donor parent was scored as ‘B,’ indicating a genotypic resemblance of IRBB60 variety. However, progenies that followed heterozygous banding pattern were scored as ‘H,’ indicating a genotypic resemblance of both parents.

### 4.4. Foreground and Background Selections

Six linked markers out of 15 tested were confirmed to be polymorphic between the two parents for bacterial leaf blight resistance genes, while two linked markers tested were confirmed polymorphic between the two parents for blast resistance genes. The foreground markers were used to select only progenies that carried both *Xoo* and blast resistance genes. A total of 472 SSR markers spread across the 12 chromosomes in rice were molecularly screened for heterogeneous alleles (polymorphism). Out of the 472 screened, 79 polymorphic rice markers were identified and used for background selection. The 79 polymorphic markers were distributed across the 12 chromosomes, with each chromosome getting minimum of four polymorphic markers (Table 5).

### 4.5. Phenotypic Selection, Characterization for Agro-Morphological Traits and Data Analysis

At every stage of backcrossing, the whole population was subjected to phenotypic selection after carrying out foreground and background selections. The phenotypic selection was carefully done to ensure that only plants with the *Xoo* and blast resistance genes with clear visual phenotypic expression were selected. Agro-morphological characteristics were recorded on suitable plants for yield and yield component traits following a standard procedure described by IRRI [43]. Data obtained on foreground marker genotyping were subjected to a chi-square test using SAS software version 9.4 and compared with Mendelian genetics. Additionally, quantitative data obtained from yield and yield component traits were subjected to descriptive statistical analysis using SAS software. Maximum RPGR was determined by subjecting the genotypic data obtained from background selection with the 79 polymorphic markers to further analysis in genetic software called Graphical Genotyper (GGT 2.0) [44].

## 5. Conclusions

This work showed that marker-assisted backcross breeding is a useful tool for introgression of resistance genes from the donor parent into the recipient. It also revealed the potentials of foreground selection in identifying the target genes for resistance of bacterial leaf blight and blast infections. The ability of background selection in recovering the recurrent parent genome was also exposed in this study. In addition, the capability of backcross breeding method to introgress the resistance genes and also reduce the donor parent genome were evident in this breeding program. The success story of this research is the recovery of the recipient parent genome in Putra-1 and its yield in addition to the introgression of bacterial leaf blight resistance. The introgression of the *Xoo* R-genes were faster through the adopted marker-assisted backcross breeding compared to what could be obtained through conventional breeding. The high percentage of recurrent parent genome recovery in this experiment is an indication of the available potentials of marker-assisted backcross breeding in recovering the genomes of the recipient parent in rice and other cereal crops. From the available records, this work presents the very first attempt to manipulate the genome of the elite Malaysia rice variety “Putra-1” for bacterial leaf blight and blast resistance without jeopardizing its high yielding characteristic. Therefore, the newly improved lines are recommended as new varieties to farmers in rice growing regions.

## Figures and Tables

**Table 1:** Foreground marker segregation analysis of the BC_1_F_1_ progenies.

Molecular Marker	Chro. No.	Marker Segregation Analysis		χ^2^ (1:1)
BLB		A	H	
Xa21FR	11	122	108	0.85
Xa13prom	8	110	125	0.96
RM13	5	130	118	0.58
MP	4	100	122	2.18
Blast				
RM8225	6	120	112	0.28
RM6836	6	120	112	0.28

d.f. = 1; χ^2^ (0.05,1) = 3.84.

**Table 2:** Foreground marker segregation analysis of the BC_2_F_1_ progenies.

Molecular Marker	Chro. No.	Marker Segregation Analysis		χ^2^ (1:1)
BLB		A	H	
Xa21FR	11	130	106	2.44
Xa13prom	8	121	106	0.99
RM21	11	130	106	2.44
MP	4	112	96	1.23
Blast				
RM8225	6	128	108	1.70
RM6836	6	128	108	1.70

d.f. = 1; χ^2^ (0.05,1) = 3.84.

**Table 3:** Foreground marker segregation analysis of the BC_2_F_2_ progenies.

Molecular Marker	Chro. No.	Marker Segregation Analysis			χ^2^ (1:1)
BLB		A	H	B	
Xa21FR	11	176	25	17	361.40
Xa13prom	8	129	57	32	135.94
RM21	11	176	25	17	361.40
MP	4	49	54	115	95.47
Blast					
RM8225	6	69	100	49	4.80
RM6836	6	69	100	49	4.80

d.f. = 2; χ^2^ (0.05,1)=5.99.

**Table 4:** Comparative agro-morphological performance of the selected improved lines.

Improved Lines	PH (cm)	FLWR	NP/H	DF	DM	NT	PL (cm)	TNG/P	1000GW (g)	TGW/H (g)	SLWR	Y/HA (t/ha)	%RPGR	P7.7	P7.2
BC_2_F_2_–157	103.01	13.46	11	72	108	11	38.56	154	78.63	57.42	3.39	9.18	97.10	HR	HR
BC_2_F_2_–122	117.01	13.20	13	78	102	15	31.96	152	81.83	53.64	3.84	8.58	94.30	R	HR
BC_2_F_2_–9	110.51	12.24	15	73	106	17	30.06	137	82.83	59.28	3.65	9.48	94.00	R	HR
BC_2_F_2_–196	109.21	9.74	20	75	103	18	34.06	172	76.33	56.28	4.10	9.00	96.00	HR	HR
BC_2_F_2_–120	114.71	13.06	16	74	105	14	34.36	177	81.93	60.70	3.88	9.71	96.30	R	HR
BC_2_F_2_–208	102.21	11.49	17	79	109	18	29.56	142	80.63	44.47	3.93	7.11	93.30	R	HR
BC_2_F_2_–155	117.51	11.66	15	77	110	17	29.66	136	78.63	45.12	3.82	7.22	97.90	R	HR
BC_2_F_2_–4	119.81	17.55	22	76	104	26	30.16	196	79.53	49.90	4.18	7.98	98.70	HR	HR
BC_2_F_2_–109	115.71	13.32	17	72	107	15	32.56	166	76.83	46.06	3.99	7.37	93.20	R	HR
BC_2_F_2_–161	112.51	12.14	14	79	107	16	32.66	207	82.53	63.98	3.96	10.23	95.60	R	R
BC_2_F_2_–144	114.01	15.03	13	77	102	12	33.76	203	77.93	43.61	4.04	6.98	95.80	R	HR
BC_2_F_2_–1	118.51	11.08	20	75	105	17	31.96	205	81.33	41.51	3.75	6.64	96.90	R	HR
BC_2_F_2_–50	96.51	15.28	16	74	109	18	34.26	209	77.33	73.83	4.21	11.81	94.10	R	HR
BC_2_F_2_–172	106.51	16.30	13	76	106	15	30.86	211	80.63	48.17	2.93	7.70	96.10	R	HR
BC_2_F_2_–166	104.01	9.74	14	75	103	17	34.06	172	76.33	56.28	4.10	9.00	96.90	R	R
BC_2_F_2_–14	108.71	15.03	13	77	102	11	33.76	203	77.93	43.61	4.04	6.98	98.50	R	HR
**Mean**	110.65^a^	13.14^a^	15.94^a^	75.75^a^	105.8 ^a^	16.06 ^a^	32.64 ^a^	177.6 ^a^	79.45 ^a^	52.74 ^a^	3.86 ^a^	8.44 ^a^	95.9		
SE	±1.13	±0.48	±0.84	±0.66	±0.66	±0.89	±0.62	±7.40	±0.54	±2.32	±0.08	±0.37			
	116.50^b^	12.56^b^	15.00 ^b^	85.67^b^	120.67 ^b^	15.00 ^b^	31.83 ^b^	148.00 ^b^	75.53 ^b^	50.41 ^b^	3.92 ^b^	8.07 ^b^			
**Recurrent Parent**															
SE	±1.40	±0.97	±0.58	±1.45	±0.88	±0.58	±1.05	±3.61	±0.75	±1.86	±0.11	±0.30			

**Note**: PH = plant height, FLWR = flag-leaf length:width ratio, NP/H = number of panicles per hill, DF = days to 50% flowering, DM = days to maturity, NT = number of effective tillers, PL = panicle length, TNG/P = total number of grains per panicle, 1000GW = one thousand grain weight, TGW/H = total grain weight per hill, SLWR = seed length:width ratio, Y/HA = yield per hectare, %RPGR = percentage recurrent parent genome recovery, P = pathotype. a,b: Values that follow the same alphabets are statistically the same (*p* > 0.05) while values that follow different alphabets are statistically different (*p* < 0.05) from each other.

**Table 5:** SSR markers used for background selection.

S/n	Name of Polymorphic SSR Markers Identified	Chro. No. Position
1	RM313, RM309, RM463, RM7376, RM117, RM28076, RM1261, RM415	12
2	pTA248, Xa21FR, RM6293, RM206, RM21	11
3	RM1375, RM294A, RM333, RM375	10
4	RM23865, RM410, RM342, OSR28, RM219, RM160	9
5	RM547, RM447, RM6208, RM25, RM310, RM544, RM5556, RM3761, Xa13prom	8
6	RM72, RM336, RM1134, RM10, RM432, RM1973	7
7	RM588, RM508, RM6836, RM8225, RM402	6
8	RM13, RM1089, RM1237, RM305, RM233A, RM1253, RM122	5
9	MP, RM518, RM8213, RM241, RM127, RM3843, RM261	4
10	RM1, RM520, SSR21, RM6308, RM232, RM130	3
11	RM262, RM525, RM573, RM452, RM250, RM5390, RM561, RM211, RM3248	2
12	RM431, RM272, RM302, RM10025, SSR23, SSR13, SSR26	1
